# Effect of Botulinum Toxin Injection into the Masseter Muscle in Patients with Bruxism: A single-arm, Institutional-Based Prospective Clinical Study

**DOI:** 10.4317/jced.62496

**Published:** 2025-03-01

**Authors:** Shehab Ahmed Hamad, Osama Ahmed, Ahmed Alraad

**Affiliations:** 1 Professor of oral and maxillofacial surgery, Kurdistan Higher Council of Medical Specialties, Ziraah square, Erbil, Iraq; 2 Medical student, Carol davila University of Medicine, Bucharest, Romania

## Abstract

**Background:**

The aim of this study was to evaluate the effect of botulinum toxin injection into the masseter muscle in patients with bruxism, focusing on pain, maximum mouth opening, occlusal bite force, and masseter muscle thickness.

**Material and Methods:**

Thirty patients with bruxism (21 females, 9 males; mean age 32 ± 10 years) were injected with 10 IU of botulinum toxin into each masseter muscle. Evaluations were conducted at baseline and at 4, 8, and 12 weeks post-injection to assess pain, maximum mouth opening, occlusal bite force, and masseter muscle thickness.

**Results:**

Pain scores significantly declined from 6.8 ± 1.2 to 3. 4± 1.1 at 4 weeks. Mouth opening showed a significant improvement, increasing from 38.2 ± 3.9 mm to 41.3 ± 4.0 mm at 4 weeks. Bite force was significantly reduced from 500.5 ± 45.2 N to 422.0 ± 39.2 N at 4 weeks. Masseter muscle thickness also significantly decreased, from 13.4 ± 1.6 mm to 11.8 ± 1.5 mm at 4 weeks. A positive correlation was observed between the reduction in masseter muscle thickness and the decrease in bit force.

**Conclusions:**

Botulinum toxin injection was safe and effective in reducing pain, masseter muscle thickness, and bite force, as well as in improving mouth opening in patients with bruxism.

** Key words:**Bite force, botulinum toxin-A, bruxism, masseter muscle, mouth opening.

## Introduction

Bruxism is the most commonly encountered non-functional movement disorder associated with the muscles of mastication, where sufferers involuntarily and unnecessarily clench or grind their teeth. This disorder can occur during sleep, known as sleep bruxism, or while the individual is awake, termed awake bruxism. It often leads to various issues, including headaches, temporomandibular disorders, muscular pain and strain, and dental problems such as tooth structure loss, fractures, and damage to dental restorations (1).

Determining the prevalence of bruxism presents challenges, primarily due to the condition’s variable manifestations among individuals and the complexities involved in distinguishing abnormal function from normal physiological activity. According to the most extensive epidemiological study conducted Maluly *et al*. (2), the prevalence within the general population ranges from 5.5% to 7.4%, depending on the criteria applied.

The aetiopathophysiology of bruxism is not well understood; however, is thought to be multifactorial, involving dental, physiological, psychological, and neurological factors (3). A significant consensus exists among researchers that the primary trigger of bruxism symptoms originates in the central nervous system. Disturbances in the catecholamine levels, particularly dopamine, which influence mandibular motor dysfunctions, are considered crucial in the underlying mechanisms of bruxism (4).

Due to the complex and multifactorial nature of bruxism, no single treatment standard can be universally applied. Among various management strategies, stabilization splint is widely regarded as the gold standard in the existing literature (5). However, the choice of treatment may vary based on the underlying causes of bruxism, leading to the consideration of various approaches for each patient, including pharmacotherapy, physiotherapy, behavioral interventions, botulinum toxin injections, and stabilization splints (6). 

 Botulinum toxin type A (BTX-A), a neurotoxin derived from Clostridium botulinum, has gained attention as a promising treatment for bruxism and myofascial pain dysfunction syndrome. This neurotoxin permanently attaches to presynaptic cholinergic receptors and competitively inhibits the neurotransmitter acetylcholine, leading to temporary muscle paralysis (7).

The masseter muscle, a pivotal component in the process of mastication, is often the target of BTX-A treatment for bruxism and myofascial pain dysfunction syndrome. This study aimed to assess the efficacy of BTX-A injection into the masseter muscle in alleviating the symptoms and consequences of bruxism.

## Material and Methods

-Study Design, Setting, Ethical Issues, and Sample Size Calculation

This study employed a single arm prospective interventional design with repeated measures. The sample consisted of patients attending a university dental clinic who sought treatment for bruxism between November 2022 and August 2024. The institutional ethical committee reviewed and approved the study (No. 235, date 12.10.2022). The purpose of the study was explained to the patients, and an informed consent was obtained. The sample size was calculated using the G*power software. Based on previous studies and a power analysis targeting 80% power with an α- level of 0.05, a sample size of 30 participants was selected.

The inclusion criteria were age over 18 years diagnosis of bruxism in accordance with the International Classification of Sleep Disorders Revised (ICSD-R) criteria.8, and presence of hypertrophy of the masseter muscle, confirmed by clinical examination and ultrasound (US) measurement.

The exclusion criteria were history of surgery or trauma to the jaw or masseter muscle, presence of other temporomandibular joint disorders unrelated to bruxism (e.g., arthralgia, disc displacement disorders, degenerative joint disease, subluxation), pregnant or breastfeeding women, known allergy to BTX-A or albumin, neuromuscular disorders, such as amyotrophic lateral sclerosis, motor neuropathy, myasthenia gravis, or Lambert-Eaton syndrome, use of medications that affect neuromuscular transmission (e.g., benzodiazepines), missing posterior teeth, use of a removable dental prosthesis or undergoing orthodontic treatment, and use of any type of occlusal splint.

-BTX-A Injection:

BTX-A (BOTOX®, Allergan Pharmaceuticals, Turkey) was utilized in this study, with 10 IU of BTX-A injected at three sites in each masseter muscle. The first injection was administered approximately 1 cm below the zygomatic arch and 1 cm anterior to the posterior border of the mandibular ramus. The second injection was located midway between the zygomatic arch and the angle of the mandible, also 1 cm anterior to the posterior border of the mandibular ramus. The third injection site was positioned approximately 1 cm above the angle and 1 cm anterior to the posterior border of the mandibular ramus. Following skin preparation with antiseptic, the injection sites were identified by palpating the masseter muscle during clenching. The drug was injected slowly and deeply at the predetermined points, after aspiration, using a 30-gauge needle attached to a 1 ml syringe, taking care to avoid the parotid gland and facial nerve. After injections, the patient was instructed to avoid massaging the area and to refrain from vigorous physical activity for the next 24 hours.

The patient was assessed at 4, 8 and 12 weeks post-injection. The following parameters were evaluated and compared to pre-injection values.

Pain: Assessed by the patient using numeric rating scale from 0 (no pain) to 10 (worst possible pain).

Mouth opening (mm): The maximum interincisal distance was measured using a ruler.

Bite force (N): Measured using bite force transducer. The patient was instructed to bite the occlusal arm with maximum force for 5 seconds, and this was repeated three times. The mean value of both sides was recorded.

Masseter muscle thickness (mm): Measured by a radiologist using ultrasound. The mean value of both sides was calculated.

-Statistical Analysis

Data were analyzed using SPSS software for Windows (ver. 24.0; IBM, Armonk, NY, USA). Descriptive data were expressed as mean ± standard deviation (SD). The Shapiro-Wilk test was employed to assess the normality of data distribution. A one-way ANOVA was utilized for the comparison of parameters with normal distribution, while Friedman test was used for parameters that did not meet normality assumptions.

For post-hoc statistical analysis, paired t-tests with Bonferroni correction were applied after one- way ANOVA, and the Nemenyi test was employed following the Friedman test. The Spearman correlation coefficient was calculated to assess the correlation between the reduction in masseter muscle thickness and the corresponding reduction in the occlusal bite force. A *p-value* of <0.05 was considered statistically significant.

## Results

All participants completed the three follow-up periods. The sample included 21 females and 9 males, with an average age of 32 ± 10 years (range 18-58). The outcome measures before injection and at 4, 8, and 12-weeks post-injection are presented in  Table 1, with post hoc analysis results shown in  Table 2.

The mean pain scores significantly decreased following the BTX-A injection, from 6.8 ± 1.2 at the baseline to 3. 4± 1.1 at 4 weeks, 2.1 ± 1.0 at 8 weeks, and1.6 ± 0.3 at 8 weeks (*p* < 0.001). The degree of mouth opening showed significant improvement, increasing from 38.2 ± 3.9 mm before injection to 41.3 ± 4.0 mm at 4 weeks post-injection, 43.1 ± 3.8 mm at 8 weeks, and finally to 44.6 ± 3.5 mm at 12 weeks (*p* < 0.001).

Bite force demonstrated a significant reduction, decreasing from 500.5 ± 45.2 N before injection to 422.0 ± 39.2 at 4 weeks, 360.5 ± 39.2 N at 8 weeks, and finally to 310.5 ± 41.7 N at 12 weeks (*p* < 0.001). Masseter muscle thickness consistently declined from 13.4 ± 1.6 mm at baseline to 11.8 ± 1.5 mm at 4 weeks, 10.8 ± 1.4 mm at 8 weeks, and ultimately to 10.2 ± 1.3 mm at 12 weeks (*p* < 0.001).

Spearman’s correlation coefficient analysis between reduction in masseter muscle thickness and occlusal bite force showed a strong positive correlation at 4 weeks (r = 0.65, *p* = 0.01), a moderate positive correlation at 8 weeks (r= 0.45, *p* = 0.05) and a weaker, non-significant correlation at 12 weeks (r = 0.30 *p* = 0.20).

## Discussion

The present study assessed the impact of BTX-A injections into the masseter muscle on orofacial pain, mouth opening, bite force reduction, and masseter hypertrophy in patients with bruxism. The masseter muscle was selected as the injection site due to its primary role in repetitive grinding motions characteristic of bruxism and its accessibility for injection without requiring imaging guidance or general anaesthesia (9). Targeting other muscles (e.g., temporalis, medial and lateral pterygoid, digastric, and geniohyoid) could interfere with chewing and swallowing functions and might need ultrasound guidance and general anaesthesia.

Nocturnal bruxism may lead to pain across the head, neck, jaw, teeth, and temporomandibular joint. The pain and discomfort experienced by bruxism patients primarily arises from increased muscle activity and the intramuscular Botox injection can induce temporary muscle paralysis, promoting muscle relaxation and potentially alleviating pain (10). Moreover, BTX-A may inhibit pain-related neurotransmitter release- specifically, substance *P* from the dorsal root ganglion-and reduce the transport of transient receptor potential to neuronal cell membranes (11).

 In this study, pain levels significantly declined following Botox injection, with these effects persisting throughout the evaluation periods, reinforcing the long-lasting analgesic effects of this treatment. These results align with previous findings (12). BTX-A injection was recommended as a first-line treatment for bruxism by Hosgor *et al*. (13). Additionally, Zhang *et al*. (14) found BTX-A injections to be more effective in alleviating the pain of bruxism compared to occlusal splint therapy.

A significant improvement in mouth opening was observed within the first month post-injection, which persisted through the next two months. The reduction of muscle tension after BTX-A injection likely contributed to the improvement of mandibular movement by decreasing the force exerted by spastic and hyperactive masseter muscle. The steady progression in mouth opening throughout the evaluation period suggests that BTX-A injection may support muscle elongation and the restoration of normal function. These results are in accordance with earlier studies.

Fietzek *et al*. (15) examined the effect of BTX-B injection into the masseter muscles of patients with stroke, hypoxic encephalopathy, and traumatic brain injury, noting a significant improvement in mouth opening compared to a placebo. Similarly, Shandilya *et al*. (16) observed substantial gains in mouth opening in patients with submucous fibrosis after BTX-A injection into masticatory muscles. However, in contrast to our findings, De Carli *et al*. (17) reported no significant improvement in mouth opening following BTX-A injection into the masseter muscles of patients with myofascial pain, despite effective pain reduction. Possible explanation for this discrepancy includes factors such as inadequate dosage, improper technique of injection, concurrent joint pathology, compensatory hyperactivity of other masticatory muscles and individual variations in BTX metabolism, as certain patients may metabolize the toxin more quickly.

The marked reduction in occlusal bite force observed in this study aligns with findings reported in several previous studies. The temporary weakening of the masseter muscle following BTX injection —due to the inhibition of acetylcholine release —reduces muscle contraction strength and subsequently lowers occlusal bite force. In parallel with our results da Silva Ramalho *et al*. (18) also reported a noTable decrease in maximum bite force after BTX injection into the masseter muscle and/or temporalis of patients with bruxism, particularly at 15, 90, and 120 days post-injection.

 In addition, our study recorded a significant reduction in masseter muscle thickness, echoing findings from recent studies that demonstrate BTX- induced masseter muscle atrophy. This reduction occurs due to the temporary blockage of acetylcholine, the primary neurotransmitter at the neuromuscular junction, thereby reducing muscle bulk. Various studies have documented varying degrees of reduction in the masseter muscle thickness following the application of BTX-A (19). For instance, To *et al*. (20) found a 31% decrease in masseter muscle volume three months post-BTX-A injection, using ultrasound and electromyography for assessment. Notably, six out of nine masseter muscles assessed maintained the atrophic condition. Similarly, another study utilizing ultrasound measurements reported up to 60% reduction in masseter muscle volume, with the most significant decrease occurring in the third month post-treatment.

A significant limitation of the current study is its single-arm design, lacking a control group for direct comparison. Future research should incorporate larger sample sizes, randomized controlled trials, and extended follow-up periods to assess the long-term impact of botulinum toxin on bruxism more comprehensively.

## Conclusions

Botulinum toxin type A(BTX-A) is a safe and effective treatment for reducing pain associated with bruxism, improving maximum mouth opening, and decreasing both masseter muscle thickness and occlusal bite forces. Additionally, a positive correlation was observed between the reduction in muscle thickness and bite force. These beneficial outcomes were sustained for six months post-injection, supporting BTX-A as a viable therapeutic option for managing bruxism-related symptoms.

## Figures and Tables

**Table 1:** Pain, mouth opening bite force and masseter muscle thickness after BTX-A injection.

Parameter	Baseline Mean (SD)	4 weeks Mean (SD)	8 weeks Mean (SD)	12 weeks Mean (SD)	P value
Pain score (VAS)	6.85 (1.2)	3.4 (1.1)	2.1 (1.0)	1.6 (0.3)	<0.0001
Mouth opening (mm)	38.2 (3.9)	41.3 (4.0)	43.1 (3.8)	44.6 (3.5)	<0.0001
Bite force (N)	500.5 (45.2)	422.0 (39.2)	360.5 (39.2)	310.5 (41.7)	<0.0001
Masseter muscle thickness (mm)	13.4 (1.5)	11.8 (1.6)	10.8 (1.4)	10.2 (1.3)	<0.0001

**Table 2:** Post -hoc analysis.

	Baseline vs. 4 weeks	Baseline vs. 8 weeks	Baseline vs. 12 weeks	4 vs. 8 weeks	4 vs. 12 weeks	8 vs. 12 weeks
Pain score	<0.0001	<0.0001	<0.0001	<0.0001	<0.0001	0.010
Mouth opening	0.035	0.001	0.0001	0.64	0.002	0.58
Bite force	<0.0001	<0.0001	<0.0001	<0.0001	<0.0001	0.0001
Masseter muscle thickness	0.004	<0.0001	<0.0001	0.02	0.0006	0.03

## Data Availability

The datasets used and/or analyzed during the current study are available from the corresponding author.
